# Integrating the BAN-ADHF diuretic resistance score into same day emergency care centres for heart failure management: a conceptual framework

**DOI:** 10.3389/fcvm.2025.1675804

**Published:** 2025-09-25

**Authors:** Dominic M. Alfonso

**Affiliations:** ^1^ School of Cardiovascular and Metabolic Health, University of Glasgow, Glasgow, United Kingdom; ^2^ Ateneo School of Medicine and Public Health, Pasig, Philippines

**Keywords:** heart failure, diuretic resistance, BAN-ADHF score, risk stratification, outpatient management

## Abstract

Early recognition of diuretic resistance is essential for preventing avoidable admissions and progression of acute decompensated heart failure. This Perspective synthesises current evidence and proposes a pragmatic, digitally enabled framework for embedding the Blood Urea Nitrogen, Atrial Fibrillation, N-terminal pro-B-type natriuretic peptide Acute Decompensated Heart Failure (BAN-ADHF) score into United Kingdom same-day emergency care services. Drawing on established electronic health record capabilities and the Fast Healthcare Interoperability Resources standard, we describe a three-touch-point workflow to stratify patients into low, moderate, or high-risk tiers of diuretic resistance and generate tier-specific recommendations for oral up-titration or intravenous sequential nephron blockade. Recent data demonstrating greater natriuresis without excess renal toxicity at higher score thresholds inform these protocolised actions. By integrating the automated risk stratification with tier-specific therapy, the proposed framework aims to reduce therapeutic inertia, lighten clinician workload, and align with the National Health Service mandate for digitally supported community care. Prospective evaluation is warranted to confirm clinical and economic benefits.

## Introduction

Heart failure (HF) affects an estimated 64 million people worldwide (1). In the United Kingdom alone, more than 1 million people live with HF and around 200,000 new diagnoses are made yearly, yet 80% of first diagnoses still occur during an unplanned admission (2), underscoring the urgent need for earlier, more effective decongestion.

Loop diuretics remain the cornerstone of decongestion, yet diuretic resistance (DR) develops in 22%–35% of acute decompensated HF (ADHF) admissions (3–5), leading to a two-fold increase in-hospital mortality, longer hospital stays, and persistent congestion at discharge (6, 7). Early recognition of patients with DR in order to allow prompt up-titration of diuretics before irreversible cardiorenal injury happens remains elusive (7, 8). Current bedside markers to identify DR, such as daily weight change, serum creatinine, or N-terminal pro-B-type natriuretic peptide (NT-proBNP), still fall short to predict DR reliably and timely, delaying necessary intensification of therapy (7).

The BAN-ADHF score (Supplementary Figure 1), derived from 794 multi-trial participants across multiple clinical trials, combines eight routinely available variables [serum creatinine, diastolic blood pressure, home loop-diuretic dose (furosemide equivalents), NT-proBNP, blood urea nitrogen, atrial fibrillation, systemic hypertension, and heart failure admission within 12 months] into a single risk estimate for low diuretic efficiency (9). The model achieved a high internal c-index/AUROC of 0.87 in identifying the lowest diuretic-efficiency phenogroup and 0.92 in the external DOSE/ESCAPE trial cohort, significantly outperforming individual biomarkers (all DeLong *P* < 0.001) (9). Furthermore, a more recent study demonstrated the real-world applicability and prognostic value of the BAN-ADHF score by integrating it into a diuretic dosing calculator and used it as a clinical decision–support tool, resulting in significantly more aggressive diuretic therapy for patients with higher scores, ultimately achieving greater 24-h urine output and weight loss at discharge (both *P* < 0.001); without increasing rates of acute kidney injury or hypokalaemia (10).

Same-day emergency care (SDEC) pathways in the UK now deliver initial care for 29.8% of unplanned medical attendances, discharging 82.4% without needing admission; 27% (*n* = 188) of centres already run dedicated HF pathways that deliver same-day outpatient intravenous diuretics (11). Indeed, a recent meta-analysis shows such outpatient IV-diuretic programmes halve 30-day mortality, reduce rehospitalisation, and consistently achieve ≥80% decongestion success without excess renal or electrolyte toxicity and saving bed-days and cost (12). This provides an opportunity to embed the BAN-ADHF score across ambulatory SDEC units as an objective metric needed for personalised loop-diuretic titration, further improving admission avoidance (13) and alignment with national strategy.

**Figure 1:**
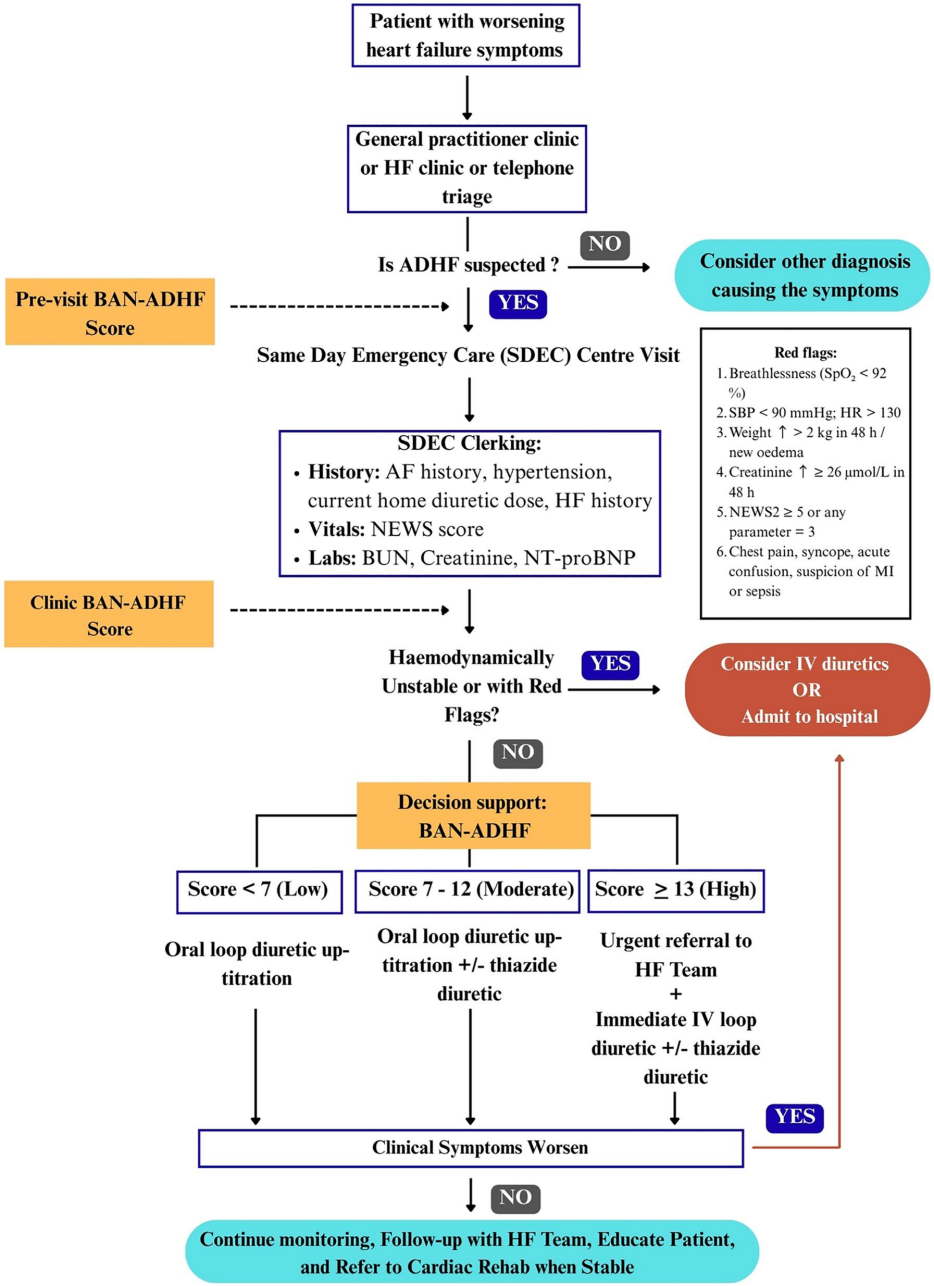
Conceptual framework of a three-touch-point workflow for integrating the BAN-ADHF score into UK same-Day emergency care (SDEC). Pre-visit: an EHR script calculates a provisional score when ≥6/8 variables are available; missing data defers scoring to clinic. Clinic: vitals, NEWS2, and same-day labs refresh the score. Red flags (14, 15) or haemodynamic instability bypass BAN-ADHF tiering and trigger immediate admission. Decision-support: evidence-based actions by risk tier—low (<7): reinforce adherence, review in 48 h; moderate (7–12): oral loop up-titration ± thiazide, review in 24–48 h; high (≥13): urgent SDEC IV loop ≥80 mg ± thiazide and HF-team review. BAN-ADHF, blood-urea-nitrogen, atrial-fibrillation, N-terminal pro-B-type natriuretic peptide acute-decompensated-heart-failure score; AF, atrial fibrillation; BUN, blood urea nitrogen; IV, intravenous; NEWS2, national early warning score 2; SDEC, same-day emergency care.

Although the clinial impact of a BAN-ADHF-directed ambulatory strategy has not yet been evaluated in prospective cohorts, our perspective is that BAN-ADHF score may help shift reactive diuretic practice into proactive, data-driven care within community SDEC units. We outline here a basic conceptual framework (Figure 1) for integrating the score into UK ambulatory HF pathways, describe the enabling general digital infrastructure and clinical workflow needed to support objective loop-diuretic titration and admission avoidance, and outline a pragmatic research agenda for future prospective evaluation.

## Workflow of BAN-ADHF in SDEC centres

To effectively integrate the BAN-ADHF score into ambulatory HF management, we propose a structured three-touch-point workflow that maps onto a typical patient journey in community care. These touch-points are: Pre-visit, clinic, and decision support. At each stage, specific processes and triggers ensure that the patient's DR risk is continuously assessed and addressed.

Pre-visit: In the UK, many HF clinics prepare for patient appointments by reviewing recent labs and vital trends via the electronic health record (EHR) (16). In this pre-visit phase, an automated BAN-ADHF score computation could be triggered. An EHR-integrated calculator would pull the latest available data for the score's variables: e.g., most recent blood chemistry (urea, creatinine), last measured NT-proBNP, presence of atrial fibrillation on record, clinic blood pressure readings, active prescription (to detect loop diuretic use), and any HF hospitalisation in the past year (from problem list or discharge summaries). However, to avoid extrapolation beyond current evidence base, the calculation is executed only when all three laboratory inputs, serum creatinine, blood urea nitrogen (BUN) and NT-proBNP have been drawn within the past 24 h (9, 10). Patients whose laboratory results fall outside the specified time window will not receive a pre-clinical score. Instead, an in-clinic score will be prompted, with all non-laboratory variables retained and applied at the time of the clinic visit. Pilot implementations of EHR-integrated HF risk calculators have shown that pulling live data into a CDS tool is not only technically possible but can also improve decision-making through predictive analytics (17). As soon as the clinician opens the patient's chart, a preliminary score <7, 7–12, or ≥13 and respective risk tier (low, moderate, high) (9, 10) would be displayed. This primes the care team by identifying patients who might require closer attention to diuresis during the visit. A patient flagged with a high preliminary score (≥13) could prompt scheduling of additional assessments (such as an on-arrival blood test or having a heart failure specialist nurse present).

Clinic: During the face-to-face clinic visit, the preliminary score is validated and refined with up-to-date clinical information. Key components at this touch-point include a focused volume status exam and point-of-care laboratory testing. For instance, if NT-proBNP was not taken within 24 h, an urgent NT-proBNP measurement should be done at the ambulatory centre. Vital signs are updated, and the clinician also verifies any changes in medications. With this new data, the BAN-ADHF score is recomputed or adjusted. In many cases, the score may remain in the same risk tier as the pre-visit estimate, but critical changes (e.g., new onset atrial fibrillation or a spike in creatinine levels) could alter the score (10). It is important to note that clinical judgment still overlays the process. For instance, if a patient has an elevated score but also has obvious non-adherence (e.g., missed diuretic doses) that can be corrected, the clinician might address adherence first. Nonetheless, the score provides an objective anchor for discussing diuretic strategy with the patient.

Decision support: After determining the patient's clinic BAN-ADHF risk tier, a decision support algorithm is applied to select the appropriate intervention. We envision three tiers with protocolised options: low risk (Score <7), moderate risk (score 7–12) and high risk (score ≥13) (10). The low-risk patients would likely demonstrate good diuretic responsiveness (10). The action here is likely modest oral loop up-titration with adherence counselling, alongside patient education on signs of fluid gain and clinical review within 48 h. The intermediate group likely has some early signs of DR (10). A possible action is to intensify oral diuretic therapy. Clinicians may up-titrate the loop diuretic dose or consider a pharmacological add-on such as a thiazide or thiazide-like diuretic if not already in place as per guidelines (14). Adding a thiazide diuretic (e.g., metolazone or Bendroflumethiazide) may be a strategy for sequential nephron blockade, helping overcome mild resistance (14, 18), with early follow-up (24–48 h) to reassess clinical status. Patients who are at high risk of diuretic failure may consequently lead to decompensation (10). The decision support here calls for a more aggressive intervention including maximising loop diuretic dose to high-dose therapy and possibly adding a thiazide diuretic or potassium-sparing diuretic (if not contraindicated) of synergistic effect on natriuresis if these medications are not yet given, but crucially, patients may immediately be referred to the HF team for IV furosemide bolus, consistent with the 2021 ESC guidelines, to achieve prompt diuresis (14). This underlines that the score could thus serve as a triage tool for intensive decongestion for such patients, rather than being sent home on oral therapy that is unlikely to succeed. Additionally, high-risk patients may trigger involvement of multidisciplinary team members such as a heart specialist and clinical pharmacist to review and optimise medications (14). The presence of clinical red flags (14, 15) or haemodynamic instability at any tier bypass the algorithm and trigger immediate escalation/admission pathways.

Indeed, integrating the BAN-ADHF score into an EHR-based CDS tool would allow delivery of standardised, risk-tailored recommendations, as was an approach shown to improve consistency of HF care in prior work (19). Clinicians can override or modify recommendations based on individual circumstances, but the tool may provide a safety net against therapeutic inertia (10). Over time, these protocols could be further refined by local audit committees, monitoring for alert fatigue and override rates. Prospective registry data will allow Bayesian updating of cut-offs, creating a learning-health-system feedback loop (20).

## Digital enablers, interoperability, and governance

Effective implementation of BAN-ADHF scoring in ambulatory care demands a robust digital infrastructure. SDEC units offer an ideal pilot environment, given their alignment with national mandates for same-day activity and comprehensive data capture (11). The Fast Healthcare Interoperability Resources Standard (FHIR) is an international specification from Health Level Seven (HL7) that defines how laboratory results, medications, and clinical observations are encoded and exchanged via secure representational state transfer (RESTful) secure web services (21, 22). In the UK, major EHR platforms such as EMIS Web®, SystmOne®, Cerner Millennium®, and Epic® already support FHIR-based integration (23). BAN-ADHF's input variables are therefore represented as native FHIR observation resources, enabling automatic unit conversions (e.g., creatinine µmol/L to mg/dl) and real-time score computation without needing extensive custom coding (22).

As clinicians open a HF patient's chart, a CDS tool embedded in the record sends a request to a FHIR end-point called BAN-ADHF score. The service first queries the regional shared care record (ShCR), an initiative of the National Heath Service (NHS) for interoperability (22, 24), to fetch the most recent laboratory measurements (i.e., within 24 h), medications, and medical history and problem code, to compute the score. The calculated score then returns to the chart as a colour-coded card displaying the recommended action tier.

Score calculations are processed under General Data Protection Regulation Article 9 (2)(h), which permits the use of sensitive health data for direct care. Each request is written to an immutable audit log reviewed quarterly by the Trust Digital Governance Board. The calculator itself runs as a containerised microapplication in the NHS Secure Cloud (IL4) (25).

## Implementation challenges and mitigation strategies

Translating this framework into practice will encounter several real-world challenges. Table 1 summarises key barriers across operational, clinical, and regulatory domains, along with proposed mitigation strategies.

**Table 1:** Anticipated implementation challenges and proposed mitigations for integrating the BAN-ADHF diuretic resistance score into ambulatory heart-failure care.

Challenge (domain)	Proposed mitigation
Workflow integration (Operational)—Additional steps for staff to compute scores and follow alerts could disrupt busy clinics (26)	Embed score calculation seamlessly into existing EHR workflow (e.g., auto-calc and alert) (26) adapted for the BAN-ADHF score
	Provide training and involve clinic staff in co-designing the process to ensure it adds efficiency (e.g., use healthcare assistants or pharmacists to gather data and prep score before clinician visit) (27)
Clinician adoption (Clinical)—Providers may be hesitant to trust a new score or alter their usual practice, worrying it undermines clinical judgement (28)	Present BAN-ADHF integration as a decision support tool (28)
	Importantly, start with a pilot where clinicians lead and refine the score and workflow. And, offer ongoing education and feedback on outcomes to build confidence in the tool
	Quantitative targets such as minimum utilisation rates, adherence rates, and override or bypass rates can be indices for an auditable approach for improvement (29)
Data accuracy & governance (Regulatory)—Ensuring all data inputs (labs, etc.) are up-to-date and consent obtained for monitoring (10, 16, 30)	Establish data verification protocols: e.g., point-of-care NT-proBNP and U&E panel performed on arrival (10)
	Use existing NHS information governance frameworks (16)

U&E, serum urea and electrolytes.

Operationally, busy clinics cannot absorb extra clicks or parallel tools to calculate a score and added steps and intrusive alerts risk slowing triage and may fragment clinical flow (26). To avoid this, the BAN-ADHF score should be calculated automatically from routinely captured fields within the existing EHR and surfaced as a light, context-appropriate prompt at clerking (26). Data collection and score preparation can be undertaken by healthcare assistants or pharmacists before the clinician visit, and an initial pilot co-designed with frontline staff should be used to refine where and how the prompt appears to make sure it does not disrupt throughput.

Clinician adoption may also pose a challenge. New risk tools may be viewed as constraining judgement, particularly if they appear prescriptive (28). The integration should therefore be framed explicitly as decision support that preserves clinical autonomy with an evident override. A clinician-led pilot, accompanied by targeted education and feedback on observed outcomes, can build confidence and calibrate expectations (28, 29). Furthermore, simple, auditable process indicators such as minimum utilisation, adherence to early review windows, and the frequency of override or bypass may provide the basis for iterative improvement without burdening teams (29).

Finally, proper implementation requires adherence to regulatory standards, up-to-date data, and compliant handling. Stale laboratory inputs undermine decision quality, and monitoring service performance requires clear governance (30). Sites should establish same-day verification protocols such as point-of-care NT-proBNP and U&E on arrival (10), align data flows with existing NHS information governance frameworks (16), and address consent where required for service monitoring (30). In practical terms, using the NHS number (England/Wales) or Community Health Index (Scotland) to deterministically link BAN-ADHF inputs (e.g., community-dispensed loop diuretics, laboratory and echocardiography results) across primary and secondary care. Privacy-preserving approaches, such as federated learning, can share model updates without moving raw patient data, especially where multi-site evaluation is planned (16). Maintaining an audit log enables transparent review of safety and service metrics and prepares the ground for a prospective evaluation (29).

By anticipating these challenges and addressing them proactively, the implementation of the BAN-ADHF score-guided care may be more seamlessly integrated into clinical pathways. Leadership support from hospital trusts and commissioning groups will also facilitate overcoming operational hurdles, by recognising this as a priority initiative aligned with broader goals of reducing HF admission and improving chronic care in the community (31).

### Discussion: future directions and a proactive approach towards diuretic management

The promise of BAN-ADHF risk-tiering will ultimately be confirmed only in prospective practice. A feasible next step is a pragmatic, cluster-randomised trial in which SDEC HF services are allocated to BAN-ADHF–guided care vs. usual practice, with days alive and out of hospital as the primary endpoint (32, 33). This may capture both clinical benefit and admission avoidance. Secondary analyses could track renal trajectories, electrolyte disturbances, health-related quality of life, and cost-effectiveness (10). Outpatient IV-diuresis programmes have already shown that such community-based trials are workable and can halve 30-day readmissions, offering a ready template for study design (13, 34).

We therefore encourage HF clinicians, informaticians, and health-systems leaders to pilot BAN-ADHF score integration, however provisionally, within routine ambulatory, SDEC HF workflows. Doing so can shift diuretic therapy from reactive rescue to proactive prevention, dovetailing with the NHS mandate for digitally-enabled, community-centred care and offering a scalable model for other healthcare systems. Early adoption, coupled with continuous audit and iterative algorithm refinement, will turn every patient encounter into an opportunity to learn, improve, and ultimately reduce the global burden of decompensated heart failure.

## Data Availability

The original contributions presented in the study are included in the article/Supplementary Material, further inquiries can be directed to the corresponding author.
