# Supplementary material for: Integrating the BAN-ADHF diuretic resistance score into same day emergency care centres for heart failure management: a conceptual framework
Source: Front Cardiovasc Med. 2025 Sep 25;12:1675804. doi: 10.3389/fcvm.2025.1675804 (PMC12508824; doi:10.3389/fcvm.2025.1675804)
Supplement: Supplementary file 1 [file Datasheet1.pdf]

Supplementary Material

Supplementary Table S1: Scoring of the BAN-ADHF score to predict the risk of diuretic resistance.

**Predictor Variable**	**Score**
Creatinine (mg/dL)	
<1.2	0
1.2–1.59	2
≥1.6	4
Diastolic Blood Pressure (mm Hg)	
≥60	0
50–59	1
<50	3
Home Diuretic (furosemide equivalent/day)	
<120	
120–249	0
≥250	3
	6
NT-proBNP (pg/mL)	
<5000	0
5000–12000	2
>12000	4
BUN (mg/dL)	
<20	0
20–39	2
≥40	3
Atrial Fibrillation	
No	0
Yes	2
Hypertension	
No	0
Yes	3
Previous HF < 12 months ago	
No	0
Yes	1
Points are summed across all categories (range: 0–23). Higher scores indicate greater probability of low diuretic efficiency.<br><br>Reference:<br>Adapted from Segar et al. *JACC Heart Failure*. 2024 Mar;12(3):508–520. doi: 10.1016/j.jchf.2023.09.029	

Abbreviations:
BUN – blood urea nitrogen
NT-proBNP – N-terminal pro-B-type natriuretic peptide
HF – heart failure
